# Insight into the Exoproteome of the Tissue-Derived Trypomastigote form of *Trypanosoma cruzi*

**DOI:** 10.3389/fchem.2016.00042

**Published:** 2016-11-07

**Authors:** Rayner M. L. Queiroz, Carlos A. O. Ricart, Mara O. Machado, Izabela M. D. Bastos, Jaime M. de Santana, Marcelo V. de Sousa, Peter Roepstorff, Sébastien Charneau

**Affiliations:** ^1^Laboratory of Biochemistry and Protein Chemistry, Department of Cell Biology, University of Brasilia, Brasilia, Brazil; ^2^Department of Biochemistry and Molecular Biology, University of Southern DenmarkOdense, Denmark; ^3^Laboratory of Host-Pathogen Interaction, Department of Cell Biology, University of BrasiliaBrasilia, Brazil

**Keywords:** Chagas disease, trypanosome, bloodstream trypomastigote, secretome, glycoprotein, phosphoprotein

## Abstract

The protozoan parasite *Trypanosoma cruzi* causes Chagas disease, one of the major neglected infectious diseases. It has the potential to infect any nucleated mammalian cell. The secreted/excreted protein repertoire released by *T. cruzi* trypomastigotes is crucial in host-pathogen interactions. In this study, mammalian tissue culture-derived trypomastigotes (Y strain) were used to characterize the exoproteome of the infective bloodstream life form. Proteins released into the serum-free culture medium after 3 h of incubation were harvested and digested with trypsin. NanoLC-MS/MS analysis resulted in the identification of 540 proteins, the largest set of released proteins identified to date in *Trypanosoma* spp. Bioinformatic analysis predicted most identified proteins as secreted, predominantly by non-classical pathways, and involved in host-cell infection. Some proteins possess predicted GPI-anchor signals, these being mostly trans-sialidases, mucin associated surface proteins and surface glycoproteins. Moreover, we enriched phosphopeptides and glycopeptides from tryptic digests. The majority of identified glycoproteins are trans-sialidases and surface glycoproteins involved in host-parasite interaction. Conversely, most identified phosphoproteins have no Gene Ontology classification. The existence of various proteins related to similar functions in the exoproteome likely reflects this parasite's enhanced mechanisms for adhesion, invasion, and internalization of different host-cell types, and escape from immune defenses.

## Introduction

Adhesion and invasion are the first stages of interaction between an obligate intracellular pathogen and its host-cell that involve cell surface molecules and secreted molecules. The released molecules from infectious microorganisms may also play a crucial role in pathogen life cycle and establishment of infection, such as host-defense evasion, migration across host-tissues, cell adhesion, cell-cell communication, differentiation, proliferation, and morphogenesis (Ranganathan and Garg, 2009; Soblik et al., 2011). Besides the classical secretory pathway (Schatz and Dobberstein, 1996), several functional proteins are released into the extracellular medium despite lacking any predicted signal peptides, thereby proving the existence of unconventional mechanisms of protein secretion in eukaryotes (Nickel and Rabouille, 2009).

The term secretome was first introduced in a bioinformatic survey of proteins secreted by *Bacillus subtilis* (Tjalsma et al., 2000). The authors defined the secretome as the subset of the proteome, which is secreted, in addition to the components of cellular machinery used for protein secretion. The characterization of the *B. subtilis* secretome using a proteomic approach showed that the genome-based prediction correctly identified ~50% of the proteins (Antelmann et al., 2001). By comparison, the secreted proteins encoded by the human genome represent approximately 30% of proteins (Skach, 2007). Nowadays the term secretome is used primarily to denote proteins secreted by cells into the extracellular region (Greenbaum et al., 2001). However, these released proteins are not only secreted proteins but also proteins that arise from other export mechanisms. Moreover, only those proteins that are stable in the extracellular medium will remain in abundance. Consequently, the best term to describe the protein content found in the extracellular proximity of a given biological system is the “exoproteome” (Armengaud et al., 2012).

The protozoan *Trypanosoma cruzi* is responsible for Chagas disease, which represents a major health public burden mostly in Latin America. It is estimated that about 6 to 7 milion people are infected (WHO, 2016). However, the epidemiological profile of the disease has changed in recent years due to migration, with thousands of patients in North America, Europe, Australia, and Japan (Gascon et al., 2010). The treatment of Chagas disease is currently based on chemotherapy, and no effective vaccine is available. The unique drugs used against Chagas disease, benznidazole, and nifurtimox, have high toxicity and the efficacy of the treatment declines with time of infection (WHO, 2016). Although their indication was until recently limited to the acute phase, recent studies agree to treat patients in the chronic phase (Lescure et al., 2010).

In order to carry out its lifecycle, the non-replicative and most infective life form of *T. cruzi*, the metacyclic trypomastigote, must cross the mammalian host's extracellular matrix, adhere to the cellular plasma membrane and can penetrate almost any nucleated vertebrate host-cell by a non-conventional endocytic mechanism involving host-cell lysosome recruitment and their fusion with the parasitophorous vacuole. It results in an obligatory stay in an acidic vacuole where the differentiation into the replicative amastigote form (first amastigogenesis) is initiated, and completed in the cytoplasm after escaping the parasitophorous vacuole (Andrade and Andrews, 2004). After replication, amastigotes can differentiate into tissue derived bloodstream trypomastigotes released into the bloodstream that can infect other vertebrate host-cell and the secondary amastigogenesis is observed or be ingested by the blood-sucking triatomine vectors (Vanhamme and Pays, 1995). In some cases, amastigotes may be generated extracellularly, and they are also able to infect cells (Mortara et al., 2008). Recently, a quantitative high-throughput proteomic and phosphoproteomic survey of *T. cruzi* axenic acidic-pH induced differentiation from trypomastigotes into axenic amastigotes provided insights into the molecular mechanisms coordinating this process (Queiroz et al., 2014).

The infection involves a number of parasite secretion/excretion factors, as well the release of host-membrane binding proteins to allow infection and virulence. Some mechanisms of host-cell invasion by *T. cruzi* have been described at the ultrastructural level and the biochemical strategies involved in host-parasite interaction have also been investigated (Osuna et al., 1990; Tardieux et al., 1994; Di Noia et al., 1996; Villalta et al., 2001; Ferreira et al., 2006). These mechanisms involve an increase of host-cytosolic Ca^2+^ concentration caused by the release of intracellular deposits of the ion during the parasite invasion (Osuna et al., 1986; Rodríguez et al., 1996; Pollevick et al., 2000). After the depolymerization of actin filaments and recruitment of lysosomes toward the plasma membrane by a kinesin-dependent process, it occurs the remodeling of the host-cell cytoskeleton (Osuna et al., 1986; Yoshida et al., 1989; Tardieux et al., 1994; Rodríguez et al., 1996; Villalta et al., 2001; Tarleton et al., 2007). In the past 20 years many groups have sought to identify the *T. cruzi* components involved in host-cell invasion. The image emerging from these studies is that the parasite's penetration into the mammalian cell is a multistep process involving several molecules from the parasite and host in a series of events leading to intracellular Ca^2+^ mobilization in both organisms (Burleigh and Andrews, 1998; Yoshida and Cortez, 2008). To invade mammalian cells, metacyclic trypomastigotes employ surface glycoproteins, such as gp82, gp35/50, or gp30 (a variant from gp82 expressed in isolates deficient of gp82), mucins and trans-sialidases (TS) (Buscaglia et al., 2006; Yoshida and Cortez, 2008; De Pablos et al., 2011). These parasites can also use other components, such as secreted proteins from the SAP family (proteins rich in serine, alanine, and proline) (Baida et al., 2006). Trypomastigotes also use a series of components to traverse the extracellular matrix and invade the host-cell, such as Tc-85, gp83, Tc-1, cruzipain, oligopeptidase B (OPBTc), and prolyl oligopeptidase 80 kDa (POP Tc80) (Burleigh and Andrews, 1998; Yoshida and Cortez, 2008). For example, POP Tc80 is able to degrade native collagen fibers in rat mesentery (Santana et al., 1997) and it could be secreted despite having no secretion signal sequence (Grellier et al., 2001; Bastos et al., 2013). OPBTc is active as a dimer (Motta et al., 2012) and assists in cell invasion by generating agonist modulators of Ca^2+^ required for the recruitment and fusion of lysosomes to the parasite adhesion site (Tardieux et al., 1992). In fact, the invasion of non-phagocytic cells is an active process and the cell contact generates Ca^2+^ signaling modulating factors from the parasite and/or host-cell, however the host-cell signaling pathways activated by the protozoan have not yet been fully identified (Burleigh and Andrews, 1998; Yoshida and Cortez, 2008; Caradonna and Burleigh, 2011).

Today, high-resolution mass spectrometry-based proteomic approaches have modified the view of exoproteomes. For instance, recent secretome analyses regarding *T. cruzi* Dm28c clone metacyclic trypomastigote and epimastigote (Bayer-Santos et al., 2013) and also other members of the Kinetoplastida order (*Leishmania (Leishmania) donovani, Trypanosoma congolense* and *T. evansi*) (Silverman et al., 2008; Cuervo et al., 2009; Geiger et al., 2010) have begun to unveil the links between the high diversity of secreted molecular components and their functions. Although, metacyclic trypomastigotes and tissue derived bloodstream trypomastigotes share some morphological and biological properties, both forms display different modes of interaction with host-cells (Burleigh and Andrews, 1995). Since our interest is to better understand the infection of the vertebrate host-cell by *T. cruzi*, the present investigation is the first to perform such analysis of a *T. cruzi* Y strain tissue derived trypomastigote (bloodstream form) exoproteome. Furthermore, we performed glycopeptide and phosphopeptide enrichment techniques to have a first glimpse of the post-translational modifications (PTMs) present in secreted/excreted proteins (Bendtsen et al., 2004; Conesa et al., 2005; Poisson et al., 2007; Petersen et al., 2011).

## Materials and methods

All reagents were from Sigma/Aldrich (St. Louis, USA) unless stated otherwise.

### Trypomastigote cell culture

Trypomastigotes, Y strain, were maintained in monolayers of HeLa cells grown in Dulbecco's Modified Eagle's medium (DMEM), pH 7.4, supplemented with 5% fetal bovine serum (Sorali Biotecnologia, Campo Grande, Brazil) and 100 μg/mL gentamicin, at 37°C in an atmosphere with 5% CO_2_ (Andrews and Colli, 1982), with the medium changed daily within the initial 3 days after infection. Parasites emerged from the destroyed host-cells from the 4th day and were collected on the 5th day, and consisted of over 98% trypomastigotes.

### Incubation and processing of the exoproteome

Trypomastigotes were washed 3 times by centrifugation at 2500 × g for 10 min with DMEM, pH 7.4, without serum. Then, 1.0 × 10^9^ washed parasites were resuspended in 5 mL DMEM without serum, pH 7.4, (2.0 × 10^8^ cells/mL) as described in Queiroz et al. (2013) and incubated in 25 cm^2^ culture flasks at 37°C for 3 h, with gentle shaking every 20 min. After incubation, parasite motility was checked and the sample was collected only if the medium was not acidified and about 95% of the cells remained mobile. To remove cells after the incubation, the medium was centrifuged for 5 min at room temperature 3 times to ensure complete removal of cells and avoid mechanical cell lysis: Firstly at 2000 × g, then at 4000 × g, and finally at 6000 × g, with the supernatants transferred to new tubes after each centrifugation. After cell removal, proteins were precipitated with trichloroacetic acid (TCA, 15% v/v) for 1 h at −20°C and washed 3 times with ice-cold acetone, at 14,000 × g, 15 min, at 4°C.

### Sample preparation

From one biological sample, TCA-precipitated proteins were resuspended in 20 mM triethylammonium bicarbonate (TEAB), reduced with 20 mM dithiothreitol at 56°C for 45 min, alkylated with 40 mM iodoacetamide at room temperature in the dark for 60 min and digested overnight at 37°C with 1 μg modified trypsin (Promega, Madison, USA). After digestion, the sample was acidified to a final concentration of 0.1% trifluoroacetic acid (TFA) and desalted with homemade microcolumns of Poros Oligo R3 resin (PerSeptive Biosystems, Framingham, USA) packed (1 cm long) in p200 tips (adapted from Gobom et al., 1999). Prior to lyophilization, peptide concentration was determined by amino acid analysis using a Biochrom 30 amino acid analyser (Biochrom, Cambridge, U.K.) following the manufacturer's protocol.

### TiO_2_ affinity phosphopeptide enrichment

Phosphopeptides were enriched *in batch* by TiO_2_-affinity as described elsewhere (Jensen and Larsen, 2007) with minor modifications. Briefly, the digested and desalted sample (around 20–30 μg) was resuspended in 1 M glycolic acid in 80% (v/v) acetonitrile (ACN)/5% TFA (v/v) followed by addition of 0.3 mg of TiO_2_ beads (TitanSphere beads, GL Sciences, Tokyo, Japan) before incubation under vigorous shaking for 10–15 min. Beads were spun down and supernatant transferred to new microtubes. Addition of TiO_2_ beads to the supernatants (using 0.2 mg and, later, 0.1 mg of TiO_2_) was repeated another two times. The TiO_2_ beads from the 3 rounds of enrichment were combined and washed firstly with 80% ACN/1% TFA (v/v) and then with 10% ACN/0.1% TFA (v/v) to remove non-phosphorylated peptides bound to TiO_2_ in a hydrophilic interaction liquid chromatography (HILIC) way. Phosphopeptides were then eluted with ammonia solution (0.28%), pH 11, and lyophilized.

### ZIC-HILIC glycopeptide enrichment

The combined flow-through and wash without phosphopeptides were dried down, resuspended with 0.1% TFA, desalted and lyophilized before submitted to glycopeptide enrichment and Peptide-*N*-Glycosidase F (PNGase F) digestion as described in Mysling et al. (2010) with minor modifications. Briefly homemade Zwitterionic HILIC (SeQuant, Umeå, Sweden, 10 μm) microcolumns packed (1 cm long) in p200 tips, in analogy from (Gobom et al., 1999), were manufactured prior to enrichment. The packed microcolumn was first washed with 20 μL elution solution (5% formic acid), then equilibrated with 40 μL load/wash solution (80% ACN, 1%TFA). The sample solubilized in 10 μL load/wash solution was applied into the column using a 1 mL syringe, washed with 40 μL load/wash solution and glycopeptides were eluted with 10 μL elution solution and dried. The flow-through and wash were also collected and combined. The N-linked glycan structures from the glycopeptides were removed using 0.2 units of PNGase F in 50 mM TEAB.

### HILIC fractionation

The flow through and wash from the glycopeptide enrichment step were combined and separated in seven fractions on a TSKGel Amide 80 (Tosoh Bioscience, Stuttgart, Germany) HILIC HPLC column (length: 15 cm, diameter: 2 mm, particle size: 3 μm) essentially as described elsewhere (McNulty and Annan, 2008; Queiroz et al., 2014).

### LC–MS/MS and data analysis

Samples were analyzed by an EASY-nano LC system (Proxeon Biosystems, Odense, Denmark) coupled online to an LTQ-Orbitrap Velos mass spectrometer (Thermo Scientific, Waltham, USA). Peptides from each fraction were loaded onto a 18 cm fused silica emitter (75 μm inner diameter) packed in-house with reverse phase capillary column ReproSil-Pur C18-AQ 3 μm resin (Dr. Maisch GmbH, Germany) and eluted using a gradient from 100% phase A (0.1% formic acid) to 35% phase B (0.1% formic acid, 95% acetonitrile) for 210 min for the phosphopeptide and glycopeptide enriched fractions and 77 min for each HILIC fraction, 35 to 100% phase B for 5 min and 100% phase B for 8 min in (a total of 223 min and 90 min at 250 nL/min). After each run, the column was washed with 90% phase B and re-equilibrated with phase A. Mass spectra were acquired in positive ion mode applying data-dependent automatic survey MS scan and tandem mass spectra (MS/MS) acquisition. Each MS scan in the orbitrap (mass range of m/z of 400–1800 and resolution 60,000) was followed by MS/MS of the seven most intense ions in the LTQ. Fragmentation in the LTQ was performed by Higher Energy Collisional Dissociation and selected sequenced ions were dynamically excluded for 30 s. Raw data were viewed in Xcalibur v.2.1 (Thermo Scientific, Waltham, USA). Data processing was performed using Proteome Discoverer v.1.3 (Thermo Scientific, Waltham, USA). Raw files were generated and these were submitted to searching using Proteome Discoverer with *in house* Mascot v.2.3 algorithm against *T. cruzi* downloaded (early 2012) using Database on Demand tool (Reisinger and Martens, 2009) containing the proteins of the parasite found in UniProt/SWISS-PROT and UniProt/TrEMBL. Contaminant proteins (several types of human keratins, BSA and porcine trypsin) were also added to the database and all contaminant proteins identified were manually removed from the result lists. The searches were performed with the following parameters: MS accuracy 10 ppm, MS/MS accuracy 0.5 Da, trypsin digestion with up to 2 missed cleavage allowed, fixed carbamidomethyl modification of cysteine and variable modification of oxidized methionine as well phosphorylation of serine, threonine and tyrosine residues or deamidation of asparagine for the phosphopeptide or glycopeptide enriched fractions, respectively. Number of proteins, protein groups and number of peptides were filtered for false discovery rate (FDR) less than 1%, peptides with rank 1 and proteins with at least 2 peptides (except for the phosphopeptide or glycopeptide enriched samples) using Proteome Discoverer. Only N-glycopeptides with deamidation sites within the PNGase consensus sequence (N-X-S/T/C, X ≠ P) and phosphopeptides with p*RS* probability greater than 50% (Beausoleil et al., 2006) were considered for further analysis. ProteinCenter software (Thermo Scientific, Waltham, USA) was used to generate FASTA formatted files of groups of proteins of interest. Better annotation of the identified was acquired using Blast2GO software (http://www.blast2go.com/b2ghome) using default parameters. SignalP v.4.0 (http://www.cbs.dtu.dk/services/SignalP/) and SecretomeP v.2.0 (http://www.cbs.dtu.dk/services/SecretomeP/) softwares were used to predict proteins secreted by classical and non-classical pathways, respectively, and FragAnchor (Poisson et al., 2007) to assign predicted Glycosylphosphatidylinositol (GPI)-anchored proteins. Transmembrane helices based on a hidden Markov model (TMHMM) algorithm (http://www.cbs.dtu.dk/services/TMHMM/) was used to predict the number of transmembrane helixes in the protein sequences.

### Data availability

Mass spectrometer output files (Raw data), peptide and protein identification files (MGF and MSF files) have been deposited in a public repository—The PeptideAtlas database (http://www.peptideatlas.org/PASS/PASS00621) under the dataset Tag *Tcruzi_Exoproteome_* and database identifier *PASS00621*.

## Results

### Profiling the trypomastigote exoproteome

In the experimental conditions, for 3 h, trypomastigotes exhibited normal motility and morphology. The quantification of protein released by 1.0 × 10^9^ parasites was estimated at around 60 μg.

**Figure 1:**
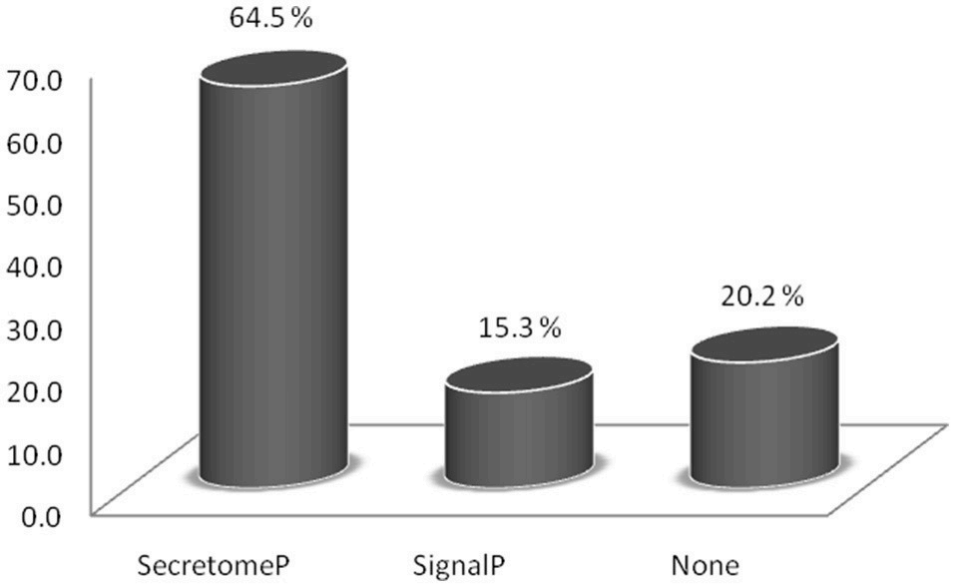
**Relative abundance of proteins identified in the trypomastigote exoproteome predicted to be secreted through classical (SignalP algorithm) and non-classical (SecretomeP algorithm) pathways**.

Taken together, the seven HILIC fractions yielded 540 protein groups identified in the trypomastigote exoproteome (Supplemental Table S1). The prediction algorithms for secretion through classical (SignalP) and non-classical (SecretomeP) pathways demonstrate that almost 80% of all identified proteins are indeed expected to be secreted, although most of them by non-classical pathways (Figure 1 and Supplemental Table S2). Only 15.3% of all identified proteins were predicted to have the signal peptide, demonstrating that just a small proportion of secreted proteins follow the classical endoplasmic reticulum/Golgi-dependent pathway.

Ninety seven proteins had a predicted Glycosylphosphatidylinositol (GPI)-anchor signal (Supplemental Table S2), being mostly TSs, mucin associated surface proteins (MASPs) and surface glycoproteins.

The TMHMM server predicted 87 proteins to have transmembrane helices, with numbers of helices ranging from 1 to 5. Most of these were members of the TS family or mucins (Supplemental Table S2). The remaining are mostly proteins known to be involved in infection, such as gp63 acid protease (mainly GPI-anchored or secreted isoforms of this protein family) (d'Avila-Levy et al., 2014) and cruzipain, which is secreted through the flagellar pocket (Murta et al., 1990). Others are known to be immunogenic proteins and glycoproteins. For example surface protein-2 is a trypomastigote-specific protein localized on the flagellum near the cell body of the trypomastigote form, as well as the putative surface protein TolT, TolT3 (Quanquin et al., 1999). And also were identified proteins with lectin domains, localized in the flagellar pocket membrane and the Golgi complex of the parasite, such as antigenic lectin-2 (Macêdo et al., 2006).

### GO classification of the trypomastigote exoproteome

From all identified proteins, 536 sequences were retrieved by Blast2GO software (Supplementary Table S2), with 48 not allocated in any GO category, most of which are hypothetical or putative proteins. Generic GO Slim was used to summarize the sub-categories of the identified protein groups.

**Figure 2:**
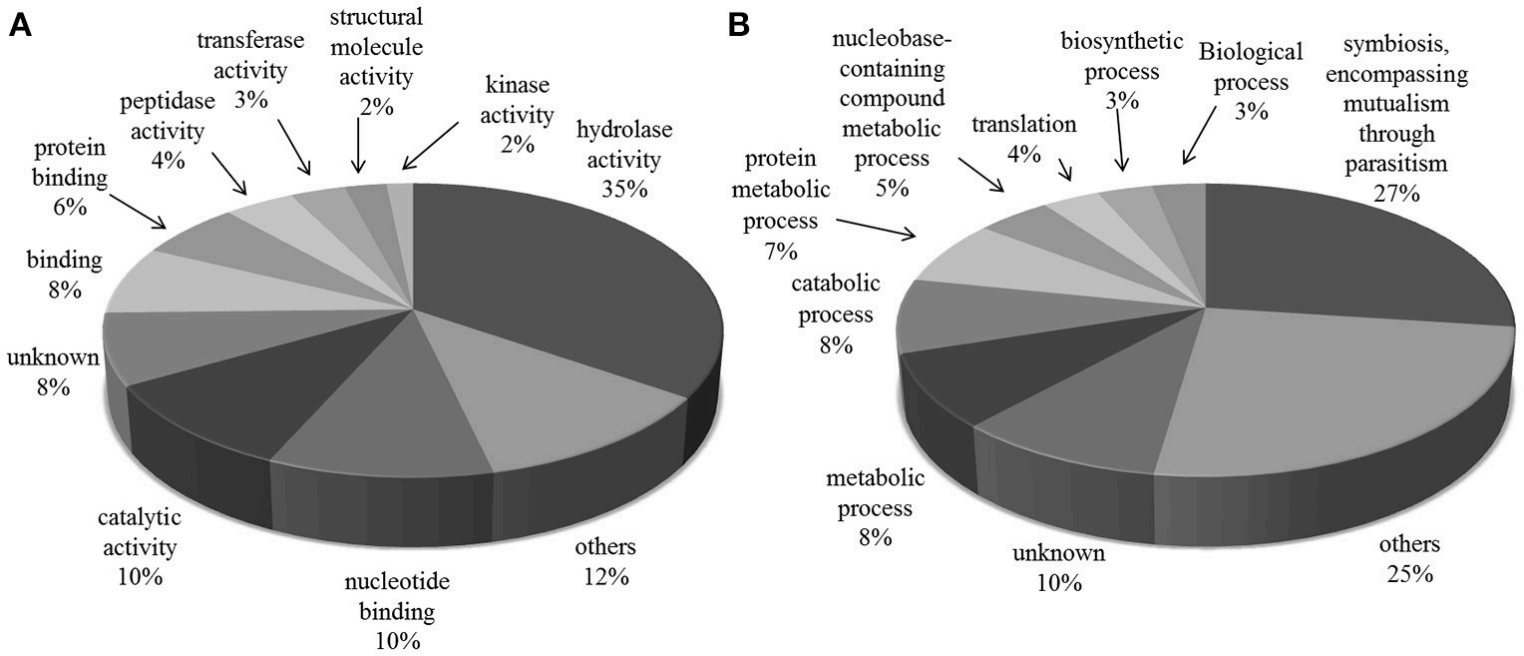
**Gene Ontology categories for all protein groups present in *T. cruzi* trypomastigote exoproteome**. Generic GO Slim was used to summarize the sub-categories. **(A)** Molecular Function and **(B)** Biological Process.

The most frequent cellular function terms identified by GO Slim were *hydrolase activity* (GO:0016787), mainly due to the large number of TS superfamily members presented in the exoproteome, “nucleotide binding” (GO:0000166) and “catalytic activity” (GO:0003824) (Figure 2).

“Symbiosis, encompassing mutualism through parasitism” (GO:0044403) was the most common GO Slim biological process term, which reflects the exoproteome's trend toward infection and host-pathogen interaction related processes. This subset of proteins, summarized in Table 1, has 229 and 4 members with predicted “hydrolase” (GO:0016787) and “transferase activities” (GO:0016740) respectively, and includes 185 members of the TS superfamily. Also along with the proteins predicted to be involved in this process, there are mucin-type glycoproteins, known to be implicated in parasite protection, attachment and the evasion of immune system (Campo et al., 2004). Other proteins found were members of FL-160 surface antigen gene family and complement regulatory proteins that inhibit the formation of the alternative and classical C3 convertase, thus preventing activation and amplification of the host-complement cascade, contributing to make trypomastigotes highly resistant to the lytic effects of vertebrate complement (Norris et al., 1997). Among the Tc13 antigen family members we identified in the trypomastigote exoproteome is the surface antigen PHGST#5 (gi|22209012|) which is supposed to act as a ligand, interacting with a neurotransmitter receptor, the beta(1)-adrenergic receptor (García et al., 2003).

**Table 1:** **Summary of proteins categorized in “Symbiosis, encompassing mutualism through parasitism”**.

**Number of proteins**	**Description (Uniprot)**	**Description (Blast2GO)**
4	85 kDa surface glycoprotein (*T. cruzi*)	trans-sialidase
1	amastigote cytoplasmic antigen (*T. cruzi*)	trans-
1	amastigote surface protein 4 (*T. cruzi*)	trans-sialidase
1	amastigote surface protein-2 (*T. cruzi*)	surface protein-2
1	c71 surface protein (*T. cruzi*)	trans-sialidase
1	complement regulatory protein (*T. cruzi*)	trans-sialidase
3	Flagellum-Associated Protein (*T. cruzi*)	trans-sialidase
4	glycoprotein 82 kDa (*T. cruzi*)	glycoprotein 82 kDa
4	putative complement regulatory protein (*T. cruzi*)	complement regulatory protein
6	putative FL-160-CRP protein (*T. cruzi*)	fl-160-crp protein
1	RecName: Full = 85 kDa surface antigen; Flags: Precursor	trans-
1	sialidase (*T. cruzi*)	trans-sialidase
2	sialidase homolog (*T. cruzi*)	trans-
1	surface antigen PHGST#5 (*T. cruzi*)	trans-sialidase
5	surface glycoprotein (*T. cruzi*)	trans-
1	surface glycoprotein GP90 (*T. cruzi*)	trans-sialidase
1	surface glycoprotein Tc-85/11 (*T. cruzi*)	trans-sialidase
1	surface glycoprotein Tc-85/16 (*T. cruzi*)	trans-sialidase
1	surface glycoprotein Tc-85/32 (*T. cruzi*)	trans-sialidase
1	surface glycoprotein Tc85-11 (*T. cruzi*)	trans-sialidase
1	surface glycoprotein Tc85-45 (*T. cruzi*)	85 kDa surface glycoprotein
2	surface protein-2 (*T. cruzi*)	surface protein-2
1	Tcc1j12.4 (*T. cruzi*)	trans-sialidase
6	trans-sialidase (*T.cruzi*)	trans-sialidase
1	trans-sialidase homolog (*T. cruzi*)	trans-sialidase
177	trans-sialidase, putative (*T.cruzi*)	trans- partial
1	trans-sialidase-like protein (*T. cruzi*)	trans-
1	trypomastigote surface glycoprotein (*T. cruzi*)	trans-
2	unknown (*T. cruzi*)	trans-

A common feature among most of the proteins we identified categorized in “Symbiosis, encompassing mutualism through parasitism” is the Laminin G domain (pfam13385). This domain belongs to the Concanavalin A-like lectin/glucanases superfamily and they are usually Ca^2+^-mediated receptors that can have binding sites for steroids, beta 1 integrins, heparin, sulfatides, fibulin-1, and alpha-dystroglycans. Proteins that contain Laminin G domains serve a variety of purposes including signal transduction via cell-surface steroid receptors, adhesion, migration, and differentiation through mediation of cell adhesion molecules (Marchler-Bauer et al., 2009).

### PTMs in secreted/excreted proteins

The glycopeptide enriched fraction yielded 27 *N*-glycopeptides encompassing 24 different glycoproteins and the phosphopeptide enrichment yielded 48 phosphopeptides from 40 different phosphoproteins (Supplemental Table S3). As a preliminary qualitative study with relatively small starting sample amount, the objective was to detect the existence of phosphorylation events on secreted/excreted proteins and, thereafter, propose which protein groups are likely to be regulated. We could observe that the majority of the identified phosphoproteins are not annotated.

**Figure 3:**
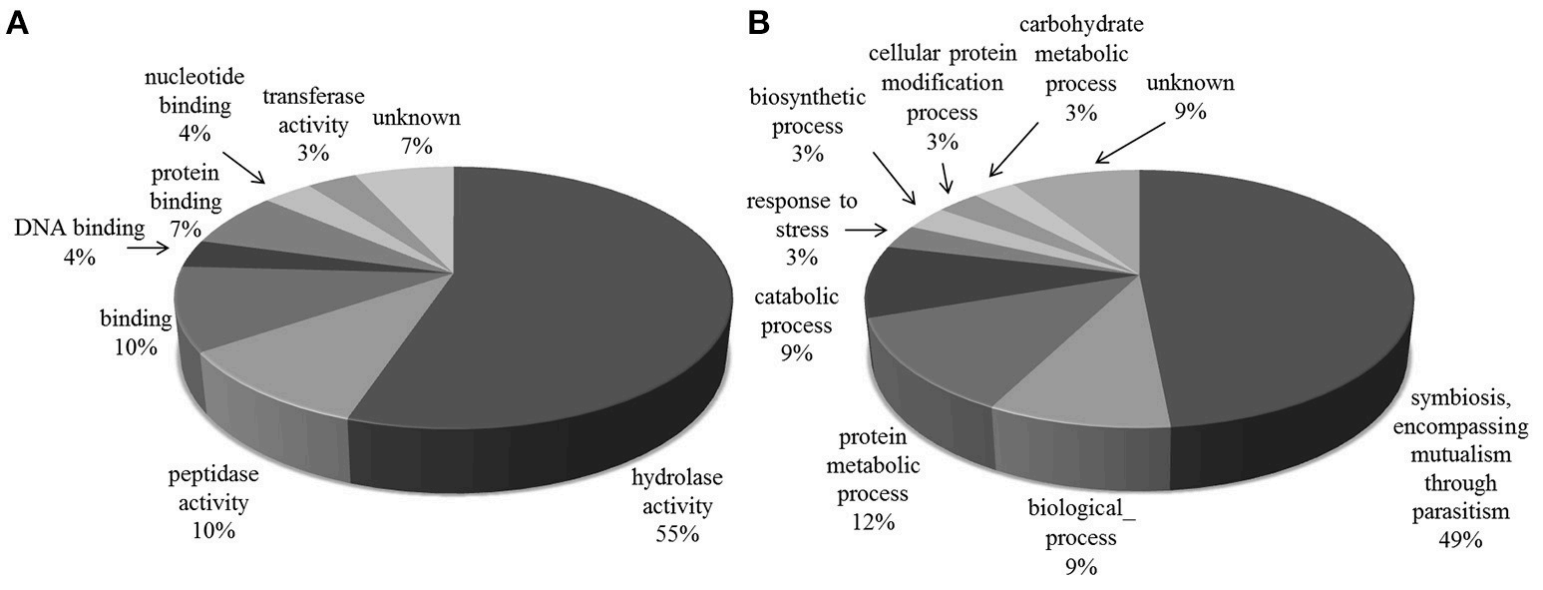
**Gene Ontology categories for N-linked glycoproteins present in *T. cruzi* trypomastigote exoproteome**. Generic GO Slim was used to summarize the sub-categories. **(A)** Molecular Function, **(B)** Biological Process.

The majority of the glycoproteins we identified in the exoproteome are TS superfamily members or surface glycoproteins and, thus, the major GO Slim function and process categories are “hydrolase activity and Symbiosis, encompassing mutualism through parasitism” (Figure 3).

## Discussion

Products secreted by cells play fundamental biological roles and represent the primary interface between parasite and host-cell. The identification of proteins involved in host-parasite interactions is important not only to understand the biological aspects of these interactions, but also to find molecules capable of blocking parasite invasion and proliferation in the host.

Bioinformatic approaches are fast and permit the prediction of secreted proteins by the presence of an N-terminal signal peptide sequence. However, there is evidence that many secreted proteins lack this signal peptide. Moreover, *T. cruzi* has polycistronic transcription, following by a trans-splicing step, and the regulation of gene expression for protein-coding genes depends entirely on post-transcriptional processes (Michaeli, 2011). Therefore, transcriptomics is limited in its ability to determine protein expression levels and post-transcriptional modifications.

The lifecycle of *T. cruzi* is complex, with several life forms and requires the expression of specialized proteins for the parasite's development and in order to circumvent the host immune response. For successful infection, *T. cruzi* needs to adapt to its new environment and choose the correct molecular tools to counteract host-defenses. For example, in mammalian hosts, bloodstream trypomastigotes try to escape the host immune system by expressing glycoproteins on their surface, while in the triatomines, they face the invertebrate host's immune system, intestinal proteases and antimicrobial compounds (Garcia et al., 2010).

Previous investigations of the *T. brucei gambiense* secretome identified 444 secreted proteins, the largest set of secreted proteins presently characterized in *Trypanosoma* (Geiger et al., 2010). And 367 proteins were reported to be released by both metacyclic trypomastigote and epimastigote *T. cruzi* life forms (Bayer-Santos et al., 2013). In this study, we found a large number of proteins released into the medium by the tissue culture cell-derived trypomastigote form (540 proteins), using stringent mass spectrometry criteria to accept protein identification (FDR <1%, only rank 1 peptide and at least 2 peptides per protein). It is noteworthy that in our study the incubation time in the serum-free culture medium was 3 h, as used for the *Leishmania (Viannia) braziliensis* secretome by Cuervo and collaborators (Cuervo et al., 2009) to limit the parasite exposure to environmental stress upon incubation conditions. This reinforces the effectiveness of this approach, which obtained a higher number of proteins in a considerably short incubation.

The SignalP and SecretomeP algorithms were used to predict whether the identified proteins were secreted by a classical pathway (with signal peptide) or a non-classical pathway. These analyses revealed that about 65% of the proteins were not secreted by the classical pathway, a small portion (15%) by the classical pathway, and the remaining with no predicted pathway, in agreement with other kinetoplastids organisms (Cuervo et al., 2009; Grebaut et al., 2009; Geiger et al., 2010). The secreted protein profile of *Leishmania (Viannia) braziliensis* promastigotes revealed that over 60% of identified proteins were predicted to be secreted, with the vast majority (about 57%) predicted as going through non-classical pathways (Cuervo et al., 2009). In the *T. cruzi* metacyclic trypomastigote and epimastigote secretomes, the secretion was predicted to be mostly via non-classical pathways (Bayer-Santos et al., 2013). Recently, a detailed proteome description of the bloodstream forms detected some proteins predicted to be secreted by non-classical secretion pathway (Brunoro et al., 2015). All together, these data indicate that protein export in lower eukaryotes was primarily by unconventional pathways, which suggests that these pathways play an important role in the release of extracellular proteins by *T. cruzi*.

To better understand the possible role of the released proteins, we used ProteinCenter and Blast2GO softwares to assign GO Molecular Function, Biological Process, and Cellular Compartment terms. Only 8 and 10% of the identified proteins could not be assigned to any molecular function and biological process, respectively. These proteins are good candidates for further characterization.

Among the identified proteins, several have been experimentally observed as released, for example the already cited TS, mucins and MASP. Glycosylphosphatidylinositol (GPI)-anchored proteins extensively coat the plasma membrane of *T. cruzi* and are involved in many aspects of host-parasite interactions, such as adhesion and invasion of host-cells, and pathogenesis (Nakayasu et al., 2009). Although they are attached to the plasma membrane by GPI anchors, enzymes such as phosphatidylinositol phospholipase C might release them from the anchors, as already reported for members of TS family (Buscaglia et al., 2006). Components of this secreted/excreted repertoire, which represent the most immunodominant antigens in *T. cruzi*, have also been employed in the development of diagnostic tests for Chagas disease (Matsumoto et al., 2002; Berrizbeitia et al., 2006). TS and mucin protein families are essential to the invasion process. They are anchored to the parasite membrane and can be shed into the bloodstream. TS bind host-cell receptors and can transfer sialic-acid residues from host glycoconjugates to the major surface glycoproteins of *T. cruzi*, mucin-like-proteins (Buscaglia et al., 2006). Also, some of MASPs have been found in the membrane of the trypomastigotes and/or secreted into the culture medium (De Pablos et al., 2011).

Likewise, the calreticulin predicted as secreted through the classical pathway, has been previously reported as a calcium binding protein that controls calcium levels in ER and has an important function in the parasite infectivity inactivating the complement component C1 (Ramírez et al., 2011, 2012).

Another protein secreted by a non-classical pathway, is superoxide dismutase. This enzyme has been suggested to be involved in parasite defense mechanisms and the establishment of trypanosome-host interaction (Kabiri and Steverding, 2001; Villagrán et al., 2005).

Furthermore, *T. cruzi* parasites excrete molecules through small vesicles at the flagellar pocket (e.g., exosomes) and larger vesicles (ectosomes) (Bayer-Santos et al., 2013). Exosomes possess a characterized mainstream protein composition (Mathivanan et al., 2010), 15 members of which we identified in the exoproteome: (i) the cytoskeletal proteins actin, cofilin 1 and tubilins; (ii) the enzymes enolase 1, PGK1, CNP, MDH 1, cyclophilin A, and peroxiredosins; (iii) the signal transduction proteins mucin 1 and 14-3-3 protein; (iv) the ATPase ATP5B; (v) ribosomal proteins and (vi) ubiquitin.

The release of such vesicles is a mode of unconventional protein secretion that has received growing attention lately. This kind of protein secretion has been correlated to autophagy pathways that do not necessarily lead to complete degradation of macromolecules (Manjithaya and Subramani, 2011). Furthermore, protein released through vesicles offers advantages such as protection against extracellular agents like proteases and antibodies, avoiding modifications that would be acquired when secreted through classical pathway and block normal protein function (Wegehingel et al., 2008). And also it has been demonstrated that these vesicles serve in cell-cell communication (Mathivanan et al., 2010; Manjithaya and Subramani, 2011), including in protozoan parasites (Regev-Rudzki et al., 2013), and remain active during starvation, when the conventional secretion is blocked (Shorer et al., 2005; Geng et al., 2010). For *T. cruzi*, extracellular vesicles promoted metacyclogenesis and also infection susceptibility of mammalian cells (Garcia-Silva et al., 2014).

In addition, this study presents the first experimental evidence of PTMs, such as *N*-glycosylation, and phosphorylation for the trypomastigote exoproteome. PTMs regulate the functional activity of proteins involved in different biological processes. Protein phosphorylation is well-recognized as a key PTM and recently, was demonstrated that phosphorylation/dephosphorylation events coordinate transformation of cell culture-derived trypomastigotes to axenic amastigotes *in vitro* (Queiroz et al., 2014). The biological relevance of the phosphorylation of released proteins remains to be explored.

In 1991, Hall and Joiner wrote: “Investigation of the molecular basis for the complex interplay between intracellular parasites and host-cells is currently underway in a number of laboratories and promises to provide exciting new insights for parasitologists, immunologists, and cell biologists” (Hall and Joiner, 1991). More than 30 years later, we could conclude that different intracellular parasites, exemplified here by *T. cruzi*, use several secretion strategies and the understanding of the recent exoproteome data on pathogenic microorganisms is a challenge, limited by experimental conditions and methods and the diversity of identified proteins. In addition, a set of identified secreted proteins with unknown functions may play important roles in *T. cruzi* invasion and, thus, there is a need for future functional characterization studies. Exoproteome analysis is still a promising area of research providing insight into *T. cruzi* infection; secreted proteins serve as a rich source of biomarkers and the development of new therapeutic strategies.

## Author contributions

Conceived and designed the experiments: RQ, CR, and SC. Performed the experiments: RQ, MM, IB, and SC. Analyzed the data: RQ, CR, MM, IB, JS, MS, PR, and SC. Contributed reagents/materials/equipments/analysis tools: JS, MS, CR, PR, and SC. Wrote the paper: RQ, CR, IB, and SC.

## Funding

This work was supported by Brazilian Grants from Conselho Nacional de Desenvolvimento Científico e Tecnológico and FAPEG and FAPDF (Fundação de Amparo a Pesquisa do Estado de Goiás e Distrito Federal) (grant no 563998/ 2010-5), CAPES Programa Nacional de Incentivo a Pesquisa em Parasitologia Básica (CAPES grant no 23038.005298/2011-83), DPP/UnB and FINEP (Financiadora de Estudos e Projetos), programme CAPES-COFECUB (723/11).

### Conflict of interest statement

The authors declare that the research was conducted in the absence of any commercial or financial relationships that could be construed as a potential conflict of interest.
